# Application of Neurotoxin-Induced Animal Models in the Study of Parkinson’s Disease-Related Depression: Profile and Proposal

**DOI:** 10.3389/fnagi.2022.890512

**Published:** 2022-05-13

**Authors:** Ya-Kui Mou, Li-Na Guan, Xiao-Yan Yao, Jia-Hui Wang, Xiao-Yu Song, Yong-Qiang Ji, Chao Ren, Shi-Zhuang Wei

**Affiliations:** ^1^Department of Otolaryngology, Head and Neck Surgery, Yantai Yuhuangding Hospital, Qingdao University, Yantai, China; ^2^Department of Neurosurgical Intensive Care Unit, Yantai Yuhuangding Hospital, Qingdao University, Yantai, China; ^3^Department of Neurology, Yantai Yuhuangding Hospital, Qingdao University, Yantai, China; ^4^Department of Central Laboratory, Yantai Yuhuangding Hospital, Qingdao University, Yantai, China; ^5^Department of Nephrology, Yantai Yuhuangding Hospital, Qingdao University, Yantai, China

**Keywords:** Parkinson’s disease, non-motor symptoms, depression, animal model, neurotoxin

## Abstract

Depression can be a non-motor symptom, a risk factor, and even a co-morbidity of Parkinson’s disease (PD). In either case, depression seriously affects the quality of life of PD patients. Unfortunately, at present, a large number of clinical and basic studies focused on the pathophysiological mechanism of PD and the prevention and treatment of motor symptoms. Although there has been increasing attention to PD-related depression, it is difficult to achieve early detection and early intervention, because the clinical guidelines mostly refer to depression developed after or accompanied by motor impairments. Why is there such a dilemma? This is because there has been no suitable preclinical animal model for studying the relationship between depression and PD, and the assessment of depressive behavior in PD preclinical models is as well a very challenging task since it is not free from the confounding from the motor impairment. As a common method to simulate PD symptoms, neurotoxin-induced PD models have been widely used. Studies have found that neurotoxin-induced PD model animals could exhibit depression-like behaviors, which sometimes manifested earlier than motor impairments. Therefore, there have been attempts to establish the PD-related depression model by neurotoxin induction. However, due to a lack of unified protocol, the reported results were diverse. For the purpose of further promoting the improvement and optimization of the animal models and the study of PD-related depression, we reviewed the establishment and evaluation strategies of the current animal models of PD-related depression based on both the existing literature and our own research experience, and discussed the possible mechanism and interventions, in order to provide a reference for future research in this area.

## Introduction

Parkinson’s disease (PD) is the most common degenerative dyskinesia disease at present. Motor retardation is the core diagnostic symptom of PD (Postuma et al., 2015). Pathologically, the progressive loss of dopaminergic neurons in substantia nigra and the formation of Lewy bodies are its main features (Ross and Bu, 2018). It has been more than 200 years since our recognition of PD (Zeng et al., 2018b), but its real etiology and pathogenesis are still unclear. The mainstream hypothesis is that PD is triggered by multiple factors including environmental, genetic, and aging factors (Fleming, 2017). Under the predicament that overemphasis on the diagnosis and treatment of motor symptoms still fails to stop the progression of the disease, people gradually realize that the non-motor symptoms (such as depression and olfactory disorder) that may manifest before the motor symptoms are potential indicators for early detection and early intervention of PD (Hasegawa, 2012). Depression, as one of the indicators, is receiving more and more attention. Similar to the effects of depression on other neurological diseases, it can cause many adverse consequences in PD patients (Menon et al., 2015) such as increasing the difficulty in the diagnosis and treatment of PD, inducing or aggravating PD symptoms, reducing patient compliance to treatment and rehabilitation, significantly increasing the rates of functional disability, recurrence, and mortality, severely reducing the quality of life of patients, and significantly increasing the economic burden on society. Although physicians and neuroscientists have been trying to solve the problem of PD-related depression, unfortunately, the relationship between depression and PD is still unclear. In the past, it was believed that depression is only a psychological stress reaction to the diagnosis of PD, just like people’s pessimistic reaction to other chronic diseases such as diabetes. However, depression actually more commonly affects PD patients than people with other chronic diseases, it shows that the rate of severe depression is twice that seen in other equivalently disabled patients (Remy et al., 2005). Especially, there is a high risk of depression in the first year after the initial diagnosis of PD (Galts et al., 2019). Therefore, it is definitely not as simple as a psychological stress reaction after the diagnosis of the disease. Of course, it is also not likely that depression occurs solely as a secondary reaction to motor deficits in PD because the natural history of depression in PD does not parallel the progression of physical symptoms (Lemke et al., 2004).

With the progress of the study, people also found that depression may be a non-motor symptom in the whole course of PD, which can occur before (in the prodromal phase), synchronous to, and after the manifestation of motor symptoms (Storch et al., 2008). A recent-year study showed that the incidence of PD is higher in people with depression than in those without depression (Schuurman et al., 2002), which suggested that depression may also be a pathogenic factor of PD (Gustafsson et al., 2015). Based on these, we can see that the relationship between depression and PD is very complex. This also explains why there are big differences in the incidence (between 4% and 75%) and prevalence (between 2.7% and 90%) of PD-related depression based on epidemiological surveys (Reijnders et al., 2008). Whether depression is a premonitory non-motor symptom (Lemke et al., 2004), a risk factor (Ishihara and Brayne, 2006), or a co-morbidity of PD (Yapici Eser et al., 2017) is still unanswered at present. However, in either case, depression will lead to catastrophic consequences, as it increases the risk of developing PD and aggravates the conditions of PD patients. Therefore, early detection, diagnosis, and treatment of PD-related depression are particularly important. Especially if depression acts as a risk factor or non-motor symptom in the prodromal period of PD, we could stretch its potential as a biological marker to assist diagnosis, guide intervention, and predict prognosis before the occurrence of motor impairment, in order to maintain the health of patients.

Unfortunately, the reality is not optimistic. At present, a large number of clinical and basic studies are focused on how to better prevent and treat depression secondary to or combined with PD. Therefore, it is difficult to really achieve the early detection, diagnosis, and treatment. Why is there such a dilemma? The lack of systematic and standardized establishment and application of the animal model of PD-related depression may be an important reason. As we all know, the utilization of PD animal models has been very mature, especially the neurotoxin-induced model (Schober, 2004; Zeng et al., 2018a), and more and more studies have found that many non-motor symptoms similar to clinical patients, including depression-like behavior, can be seen in neurotoxin-induced PD animal models (Faivre et al., 2019). Therefore, it is worth thinking whether the neurotoxin-induced PD model can be used directly or after modification in the study of PD-related depression. This article comprehensively reviewed the general depression-like phenotype of PD animal models induced by different neurotoxins, and summarized the possible mechanisms and effective intervention measures, in order to provide new ideas for the study of PD-related depression.

## 1-Methyl-4-Phenyl-1,2,3,6-Tetrahydropyridine (MPTP) And PD-Related Depression

The induction of Parkinsonian-like symptoms by MPTP was discovered in the 1980s from the misuse of defective chemosynthetic drugs by drug users (Schintu et al., 2012). Since then, with the in-depth study of the role of environmental toxins in the development of PD, MPTP has received widespread attention as an experimental tool to simulate PD, especially in the establishment of PD animal models. Non-human primates and mice are common MPTP-induced PD model animals, and other models using rats, guinea pigs, and miniature pigs have also been reported. However, it is worth noting that rats are not sensitive to MPTP due to the limited ability of transforming MPTP to its active form 1-methyl-4-phenylpyridium (MPP^+^). Therefore, rats should be induced directly with MPP^+^for the modeling of PD. In general, MPTP is mostly administered by intraperitoneal injection (Airavaara et al., 2020), and of course, there are other routes including subcutaneous injection, intramuscular injection, intravascular injection, tube feeding, nasal dripping, and brain stereotactic injection. According to the dose and frequency of intraperitoneal injection of MPTP, the MPTP-induced PD models can be divided into three types: acute, subacute, and chronic (Jackson-Lewis and Przedborski, 2007). In the acute model, MPTP was administered at 15–20 mg/kg every 2 h for four times. In the subacute model, the animals were treated with 25 mg/kg MPTP per day for five consecutive days. In the chronic model, also called the “progressive model”, MPTP was given at 25 mg/kg every 3.5 days for 5 weeks, with the addition of Probenecid before each injection to increase the abundance of MPTP in the brain. Each type has its own pros and cons, and the phenotypes varied with the type and age of animals. In either type, most animal studies simulated the motor impairment symptoms of PD, but non-motor symptoms, especially depression-like behavior, were still inconclusive. With the continuous attempts at modification of the MPTP induction method, some studies have developed potential MPTP-induced animal models that could be used for the study of PD-related depression.

### General State of PD-Related Depression Model Induced by MPTP

As summarized in Table 1, the administration route of MPTP in PD-related depression models included intraperitoneal injection (Krupina et al., 2000, 2002; Krupina et al., 2006, 2010; Pankova et al., 2004; Khlebnikova et al., 2009a, b, Khlebnikova et al., 2013; Chung et al., 2015; Zhang et al., 2015, 2016; Li et al., 2018; Sampaio et al., 2018; Kiselev et al., 2019; Okano et al., 2019; Tang et al., 2020; Yan et al., 2020), intranasal injection (Castro et al., 2013; Schamne et al., 2018), and stereotactic injection (Santiago et al., 2010; Barbiero et al., 2014; Moretti et al., 2015; Cunha et al., 2017), of which intraperitoneal injection was the main route. However, there were differences in the dose and time of MPTP administration. Notably, the neurotoxic effects of MPTP seem to be mediated by its active oxidative product MPP^+^. In addition, it was also reported that MPP^+^ was directly used to induce PD-related depression (Moretti et al., 2015; Cunha et al., 2017). It is worth noting that in the MPTP-induced model of PD-related depression, some only showed depression behavior without causing motor damage (Castro et al., 2013; Moretti et al., 2015; Cunha et al., 2017), which well simulated PD-related depression that occurs as a premonitory symptom to motor symptoms. Depression-like behaviors in MPTP-induced PD models were mostly evaluated by a set of classical animal behavior tests, such as the learned helplessness paradigm (Winter et al., 2007), including the forced swimming test (FWT), and tail suspension test (TST), which mainly manifested a helpless state of animals. Additionally, the splash test (ST) and the sucrose preference test (SPT), which were performed as an indicator of anhedonia (Matheus et al., 2016) also can be considered using. The brain regions studied were mainly the hippocampus, frontal cortex, and striatum. In particular, some studies reported the incidence of depression in the MPTP-induced PD model. For example, Tang et al. injected MPTP (30 mg/kg/day for 7 days) into 6–8-week old male C57BL/6 mice, and found about 60% of mice showed depression-like behavior (Tang et al., 2020), while Zhang et al. (2015) reported depression behavior in 66.7% of 22–26 g C57BL/6 mice after injection of MPTP (20 mg/kg/day for 7 days). Dissimilarly, some studies performed on MPTP models show no depressive symptoms in the SPT (Vucković et al., 2008; Gorton et al., 2010), FST (Santiago et al., 2010), and TST (Gorton et al., 2010). Another issue is that most MPTP-induced PD-related depression models used male rodents, whereas Schamne et al. induced PD model by intranasal administration of MPTP (1 mg/nostril) to female C57BL/6 mice of different ages (some underwent ovariectomy) to investigate the effects of gender and age (which actually reflect different hormone levels) on PD-related depression. It was found that adult female mice treated with MPTP were more likely to develop PD-related depression, and the manifestations were more diverse (such as easy to despair, anhedonia, and apathy; Schamne et al., 2018). Interestingly, anxiety-like behavior and cognitive memory impairment could also be seen in the MPTP-induced PD-related depression model (Cunha et al., 2017; Li et al., 2018). And PD models with the coexistence of pain and depression were also reported (Krupina et al., 2002, 2010).

**Table 1:** Summary of animal models of PD-related depression induced by MPTP.

Number	Reference	Model animal	Gender	Route	Dosage of MPTP/MPP+	Assessment of depression behavior
1	Zhang et al. (2016)	8-week 24–26 g C57BL/6 mice	Male	i.p.	MPTP (25 mg/kg) once a day for five consecutive days	FST↑, TST↑
2	Chung et al. (2015)	8–9-week 18–25 g C57BL/6 and ICR mice	Male	i.p.	MPTP (20 mg/kg) every 2 h for three times in one day	TST↑
3	Okano et al. (2019)	8–10-week 20–27 g C57BL/6J mice	Male	i.p.	MPTP (16, 17.5, 19, 20 mg/kg) every 2 h for four times in one day	TST↑
4	Sampaio et al. (2018)	90-day 22–30 g C57BL/6 mice	Male	i.p.	MPTP (20 mg/kg) every 2 h for four times in one day	FST↑
5	Yan et al. (2020)	C57BL-6 mice	Male	i.p.	MPTP (30 mg/kg) once a day for five consecutive days	TST↑, ST↓
6	Tang et al. (2020)	67#x02013;8-week C57BL/6 mice	Male	i.p.	MPTP (30 mg/kg) once a day for seven consecutive days	TST↑, FST↑, SPT↓
7	Zhang et al. (2015)	22–26 g C57BL/6 mice	Male	i.p.	MPTP (25 mg/kg) once a day for seven consecutive days	SPT↓, FST↑, TST↑
8	Krupina et al. (2000)	320–380 g albino Wistar rats	Male	i.p.	MPTP (20 mg/kg) once a day for 14 consecutive days	SPT↓, FST↑
9	Pankova et al. (2004)	380–430 g Wistar rats	Male	i.p.	MPTP (20 mg/kg) once a day for 13 consecutive days	/
10	Krupina et al. (2006)	320–450 g Wistar rats	Male	i.p.	MPTP (20 mg/kg) once a day for 14 consecutive days	SPT↓, FST↑
11	Khlebnikova et al. (2009a)	400–450 g Wistar rats	Male	i.p.	MPTP (20 mg/kg) once a day for 14 consecutive days	SPT↓, FST↑
12	Khlebnikova et al. (2013)	340–400 g Wistar rats	Male	i.p.	MPTP (20 mg/kg) once a day for 14 consecutive days	SPT↓, FST↑
13	Khlebnikova et al. (2009b)	320–450 g Wistar rats	Male	i.p.	MPTP (20 mg/kg) once a day for 14 consecutive days	SPT↓, FST↑
14	Kiselev et al. (2019)	20–22 g outbred mice, 24–26 g inbred C57BL/6 mice, 250–280 g outbred albino rats	Male	i.p.	MPTP (30 mg/kg)	FST↑
15	Li et al. (2018)	8-week C57BL/6 mice	Male	i.p.	MPTP (25 mg/kg) once a day for five consecutive days	SPT↓, FST↑, TST↑
16	Krupina et al. (2010)	350–450 g Wistar rats	Male	i.p.	MPTP (20 mg/kg) once a day for 2 weeks	SPT↓, FST↑
17	Krupina et al. (2002)	350–450 g albino Wistar rats	Male	i.p.	MPTP (20 mg/kg) once a day for 16 consecutive days	SPT↓, FST↑
18	Schamne et al. (2018)	3-month 20–25 g C57BL/6 mice (some were ovariectomized at 2 months old) and 20-month 25–30 g female C57BL/6 mice	Male Female	bilateral nasal drip	MPTP (1 mg/nostril)	SPT↓, ST↓, TST↑, FST↑
19	Castro et al. (2013)	4-month 300–350 g Wistar rats, 25–30 day Wistar rats	Male	bilateral nasal drip	MPTP (1 mg/nostril)	FST↑, SPT↓
20	Barbiero et al. (2014)	3-month 280–320 g Wistar rats	Male	stereotactic injection (bilateral SN)	MPTP (100 μg/site)	FST↑
21	Santiago et al. (2010)	280–320 g Wistar rats	Male	stereotactic injection (bilateral SN)	MPTP (100 μg/site)	ModifiedFST↑, SPT↓
22	Moretti et al. (2015)	3-month 30–35 g C57BL/6 mice	Male	stereotactic injection (bilateral SN)	MPP+ (8 μg/site)	TST↑, ST↓
23	Cunha et al. (2017)	60–75-day 25–35 g C57BL/6 mice	Male	stereotactic injection (bilateral SN)	MPP+ (1.8 μg/mouse and 18 μg/mouse)	FST↑, TST↑, ST↓

*i.p., intraperitoneal injection; SN, substantia nigra; FST, forced swimming test; TST, tail suspension test; SPT, sucrose preference test; ST, spatter test. ↑, The increased immobility time;↓, The presence of anhedonia*.

### Possible Mechanism of PD-Related Depression Induced by MPTP

Current research hotspot of the mechanism of PD-related depression in the MPTP-induced PD model lies in whether depressive behavior is accompanied by changes in neurotransmitters, neurotrophic factors, neuroinflammatory factors, oxidative stress, nerve regeneration, and synaptic plasticity. However, there have been few reports of altered signaling pathways, one of which is the PKA-CREB pathway (Zhang et al., 2016). The involvement of the mTOR-regulated VTA-mPFC (Ventral tegmental area-medial prefrontal cortex) neural loop on PD-related depression in the MPTP-induced mouse model has been reported (Tang et al., 2020), which for the first time verified the neural loop hypothesis in PD-related depression using PD animal model. Another research team explored the electrophysiological mechanism of MPTP-induced PD-related depression (Pankova et al., 2004). In addition, abnormal peripheral blood granulocyte count (suggesting changes in anti-inflammation and immune status) might play an important role in the development of PD-related depression (Krupina et al., 2000). Some studies also revealed that the changes in prolyl endopeptidase and dipeptidyl peptidase IV activities were also important influencing factors of PD-related depression (Bastías-Candia et al., 2019; Khlebnikova et al., 2009a).

### Possible Interventions in PD-Related Depression Induced by MPTP

At present, therapeutic intervention against depression in MPTP-induced PD models mainly includes transmitter supplementation, anti-inflammation, anti-oxidation, promoting nerve regeneration, etc., and most of the drugs used are “traditional” drugs. Among them, the use of lipid-lowering agents has been mentioned by several studies. A nasal drip of Atorvastatin could improve the MPTP-induced depression behavior by increasing the levels of nerve growth factors in the striatum and hippocampus (Castro et al., 2013). Simvastatin treatment alleviated hippocampal nerve inflammation and to some extent reversed the depression symptoms of PD mice (Yan et al., 2020). In addition to statins, Fenofibrate, a fibrate type of lipid-lowering drug, has been found to have antidepressant effects in MPTP-induced PD Wistar rats (Barbiero et al., 2014). At present, among the widely used antidepressants, only Fluoxetine has been used in the treatment of MPTP-induced PD-related depression (Zhang et al., 2015), and its antidepressant effect was not achieved through the classical pathway, but by affecting the expression of the 5-HT2B receptor on astrocytes. The role of Praxol, an antidepressant drug recommended in the clinical guidelines, on MPTP-induced PD-related depression was only mentioned when compared to the antidepressant effect of Selegiline, a monoamine oxidase B (MAOB) inhibitor (Okano et al., 2019). In addition, it was found that 1-[2-(4-Benzyloxyphenoxy) Ethyl] Imidazole could also improve the motor and depression symptoms of MPTP-induced mice by inhibiting MAOB (Chung et al., 2015). Other drugs, such as Crocin (Tang et al., 2020), dopamine D1 receptor agonist (Zhang et al., 2016), Pioglitazone (Barbiero et al., 2014), 7-Fluoro-1, 3-diphenylisoquinoline (Sampaio et al., 2018), Agmatine (Moretti et al., 2015), N-(5-Hydroxynicotinoyl)-L-glutamic acid (Khlebnikova et al., 2009a; Kiselev et al., 2019), benzyloxycarbonyl-methionyl-2(S)-cyanopyrrolidine (Khlebnikova et al., 2009b; Khlebnikova et al., 2013), and serum albumin (Pankova et al., 2004) also showed different degrees of anti-depressant effects in MPTP-induced PD models.

## 6-Hydroxydopamine (6-OHDA) and PD-Related Depression

6-OHDA is a neurotoxin formed by the reaction of dopamine quinone (a substance produced by the redox reaction of dopamine during oxidation) in the presence of iron. It was found in the 1960s that 6-OHDA could lead to the degeneration of the substantia nigra-striatum system. In the 1970s, 6-OHDA was successfully used to establish the first animal model of PD with substantia nigra-striatum lesions (Airavaara et al., 2020). After that, there were more studies on 6-OHDA-induced PD models *in vivo*, and all of them have achieved good results. The animals used were mainly rodents (rats, mice, and guinea pigs), cats, and primates, and the most popular method was the injection of 6-OHDA into the medial forebrain bundle (MFB), substantia nigra, and striatum. As more and more studies have confirmed that there is a window between 6-OHDA-induced substantia nigra degeneration and the appearance of dyskinesia, which emphasizes its value in the preclinical study of PD (Branchi et al., 2008).

### General State of PD-Related Depression Induced by 6-OHDA

As shown in Table 2, the administration route of 6-OHDA-induced PD-related depression models was only stereotactic cerebral injection. Besides the three common injection sites MFB, substantia nigra, and striatum, injection in the locus ceruleus (Szot et al., 2016; Sampaio et al., 2019), lateral ventricle (Chenu et al., 2007), and ventral tegmental area of the midbrain (Furlanetti et al., 2016) have also been reported. Injections can be bilateral or unilateral, and the MFB is the most common site for unilateral injection, whereas the striatum is the most common site for bilateral injections. One study compared bilateral striatum injection and unilateral substantia nigra injection (Chen et al., 2014). In terms of the choice of animals, rats were most commonly used and mice accounted for only 8 out of 45 studies. The dosage of 6-OHDA is not uniform at present. The depression phenotypes were evaluated by behavioral tests such as SPT, FST, TST, and ST, but it is worth noting that more than half of the studies (28/45) used only one test to evaluate depression-like behavioral changes, which was mostly FST (16/28). PD-related depression is more common in 6-OHDA-induced animals with motor impairment and is sometimes combined with other non-motor manifestations such as anxiety, cognitive memory impairment, olfactory disorder, and rhythm disorder. Meanwhile, it is encouraging that the study of depression as a prodrome of motor impairment is increasing (Branchi et al., 2008; Matheus et al., 2016; Szot et al., 2016; Marques et al., 2019; Sampaio et al., 2019), in which a small dose injection of 6-OHDA in locus ceruleus is worthy of attention. In addition, it is worth further investigation that some 6-OHDA-induced models developed transient hypokinesia that spontaneously remitted, but with a persistent depressant phenotype. The exploration of the influence of gender on PD-related depression has also been reported in the 6-OHDA-induced model (Sullivan et al., 2014). Interestingly, this study not only looked into the gender factor but also compared the correlations between each cerebral hemisphere and PD-related depression. Other studies on 6-OHDA induction combined life stress (Furlanetti et al., 2015; Dallé et al., 2020) suggested that stress+neurotoxin might be a better composite model for the study of PD-related depression.

**Table 2:** Summary of animal models of PD-related depression induced by 6-OHDA.

Number	Reference	Model animal	Gender	Location of stereotactic cerebral injection	Dosage of 6-OHDA	Assessment of depression behavior
1	Santiago et al. (2010)	280–320 g Wistar rat	Male	bilateral SN	6 μg/side	Modified FST↑, SPT↓
2	Matheus et al. (2016)	12–16-week Wistar rats	Male	bilateral dorsal striatum	10 μg/side	SPT↓, ST↓, FST↑
3	Kamińska et al. (2017)	290–320 g Wistar Han rats	Male	unilateral MFB	8, 12, 16 μg	SPT↓
4	Alzoubi et al. (2018)	150–200 g adult Wistar rats	/	unilateral MFB	4 μg	TST↑
5	Souza et al. (2018)	75-day 280–320 g Wistar rats	Male	bilateral SN	6 μg/side	SPT↓
6	Beppe et al. (2015)	350 g Wistar rats	Male	unilateral SN	12.5 μg	FST↑
7	Feng et al. (2020)	350–400 g Wistar rats	Male	unilateral MFB	8 μg	FST↑
8	Santiago et al. (2015)	280–320 g Wistar rat	Male	bilateral SN	6 μg/side	Modified FST, ↑ SPT↓
9	Santiago et al. (2014)	280–320 g Wistar rat	Male	bilateral SN	6 μg/side	Modified FST↑, SPT↓
10	Kumari et al. (2018)	250–270 g adult Wistar albino rats	/	unilateral MFB	10 μg	FST↑
11	Casas et al. (2011)	60–120-day 280–340 g Sprague-Dawley rats	Male	unilateral striatum	2 μg	FST↑
12	Prakash et al. (2013)	180–250 g adult Wistar rats	Male	unilateral SN	12 μg	FST↑
13	Antunes et al. (2014)	18-month 25–35 g C57BL/6 mice	Female	unilateral striatum	5 μg	TST↑
14	Yan et al. (2019)	8-week 20–25 g C57BL/6 mice	Male	unilateral MFB	4 μg	SPT↓
15	Sampaio et al. (2019)	3-month 180–220 g Wistar rats	Male	bilateral locus ceruleus	5, 10, 20 μg/side	FST↑, SPT↓
16	Szot et al. (2016)	3-month 25 g adult C57BL/6 mice	Male	bilateral locus ceruleus	5, 10, 14 μg/side	FST↑, SPT↓
17	Kuter et al. (2011)	300–360 g Wistar rats	Male	bilateral ventrolateral region of caudate nucleus	3.75 μg/side	FST↑
18	Bonato et al. (2018)	280–320 g Wistar rats	Male	bilateral SN	6 μg/side	FST↑
19	Chenu et al. (2007)	4-week 18–20 g Swiss mice	Male	bilateral lateral ventricle	10, 20, 30 μg	FST↑
20	Guo et al. (2021)	adult 220–250 g Sprague-Dawley rats	Male	unilateral SN	12 μg	FST↑, SPT↓
21	Tuon et al. (2014)	25–30 g adult C57BL mice	Male	bilateral striatum	4 μg /side	FST↑
22	Ilkiw et al. (2019)	280–320 g Wistar rat	Male	bilateral SN	3 μg /side	Modified FST↑, SPT↓
23	Hsueh et al. (2018)	8–12-week-old 200 g–300 g Sprague-Dawley rats	Female	unilateral MFB	8 μg	FST↑
24	Marques et al. (2019)	3-month 300–350 g Wistar rats	Male	bilateral dorsal striatum	10 μg/side	SPT↓, ST↓, FST↑
25	Vecchia et al. (2018)	270–300 g Wistar rats	Male	bilateral SN	6 μg/side	SPT↓
26	Silva et al. (2016)	3-month 270–300 g Wistar rats	Male	bilateral dorsal striatum	12 μg/side	SPT↓
27	Campos et al. (2013)	300–350 g young adult Wistar rats	Male	bilateral SN	8 or 6 μg/side	FST↑, SPT↓
28	Goes et al. (2014)	90-day 20–30 g C57BL/6J mice	Male	unilateral striatum	5 μg	TST↑
29	Chiu et al. (2015)	12-week C57BL/6 mice	Male	bilateral SN	4 μg/side	FST↑
30	Tadaiesky et al. (2008)	250 g-370 g Wistar rats	Male	bilateral dorsal striatum	12 μg/side	SPT↓, FST↑
31	Furlanetti et al. (2016)	250 g adult Sprague-Dawley rats	Female	bilateral ventral tegmental area of midbrain	3.6 μg/side	FST↑, SPT↓
32	Furlanetti et al. (2015)	250 g adult Sprague-Dawley rats	Female	unilateral MFB	19.8 μg	FST↑, SPT↓
33	Sun et al. (2016)	200–220 g adult Sprague-Dawley rats	/	unilateral MFB	8 μg	FST↑, SPT↓
34	Yu et al. (2020)	adult Sprague-Dawley rats	Male	unilateral striatum	20 μg	FST↑, TST↑
35	Bonito-Oliva et al. (2014)	25–30 g C57BL/6J mice	Male	bilateral dorsal striation	4 μg/side	TST↑, FST↑
36	Sinen et al. (2021)	3-month 250–300 g Wistar rats	Male	unilateral MFB	12 μg	SPT↓
37	Masini et al. (2018)	3-month 25–30 g C57BL/6J mice	Male	bilateral dorsal striatum	4 μg/side	FST↑
38	Petri et al. (2015)	220–280 g adult Wistar rats	Male	unilateral MFB	12.48 μg	FST↑
39	Branchi et al. (2008)	4-month Wistar rats	/	bilateral dorsal striatum	10.5 μg/side	FST↑, SPT↓
40	Chen et al. (2014)	280–300 g Wistar rats	Male	bilateral striatum, unilateral SN	12 μg/side of striatum; 8 μg per SN	SPT↓
41	Foyet et al. (2011)	230 ± 50 g Wistar rats	Male	unilateral SN	8 μg	FST↑
42	Dallé et al. (2020)	PND60-day 300 g Sprague-Dawley rats	Male	unilateral MFB	5 μg	SPT↓
43	Singh et al. (2017)	200–250 g Sprague-Dawley rats	Male	unilateral MFB	16 μg	FST↑
44	Sullivan et al. (2014)	250–275 g, Sprague-Dawley rats	Male, Female	left, right hemispheres (not bilateral)	4 μg	SPT↓
45	Mishra et al. (2019)	200–250 g adult Sprague Dawley rats	Male	unilateral MFB	16 μg	FST↑

*PND, postnatal Day; SN, substantia nigra; MFB, medial forebrain bundle; FST, forced swimming test; SPT, sucrose preference test; ST, spatter test; TST, tail suspension test; ↑, The increased immobility time; ↓, The presence of anhedonia*.

### Possible Mechanism of PD-Related Depression Induced by 6-OHDA

The exploration of the mechanism of PD-related depression using the 6-OHDA induction model is currently focused on the changes in nerve regeneration, neurotransmitter, oxidative stress, nerve inflammation, neuronutrition, etc. The hotspot brain area of study included the striatum, hippocampus, nucleus accumbens, lateral habenular nucleus, and ventral tegmental area, but there were still few studies on signaling pathways, including the BDNF-TrkB (brain-derived neurotrophic factor-tyrosine kinase receptor B; Tuon et al., 2014; Sun et al., 2016) and Wnt/β-catenin signaling pathways (Singh et al., 2017; Mishra et al., 2019). In the studies of 6-OHDA-induced PD-related depression, there was no report on the involvement of the neural loop. But some studies, suggested that the ipsilateral and contralateral connections between the midbrain dopamine system and the medial prefrontal cortex might play an important role (Petri et al., 2015).

### Possible Interventions in PD-Related Depression Induced by 6-OHDA

Among the many studies that discussed the prevention and treatment of PD-related depression in the 6-OHDA-induced models, a drug intervention is the most common method. Dixipramine (Kamińska et al., 2017), Piroxicam (Santiago et al., 2014), 5-HT4R agonists (Guo et al., 2021), Reboxetine (Bonito-Oliva et al., 2014), and Fluvoxamine (Dallé et al., 2020) could improve the depression symptoms in the 6-OHDA-induced models by increasing the level of relevant neurotransmitters. Etazolate (Alzoubi et al., 2018), aqueous extract of albizia leaves (Beppe et al., 2015), 1-(7-imino-3-propyl-2, 3-dihydrothiazolo [4, 5-d] pyrimidin-6(7H)-yl)urea (IDPU; Kumari et al., 2018), hesperidin (Antunes et al., 2014) and methanol extract of cottonrose hibiscus leaves (Foyet et al., 2011) could antagonize 6-OHDA-induced PD-related depression through the antioxidant stress response. Schisanhenol A (Yan et al., 2019) has been reported to improve depression symptoms in the 6-OHDA-induced PD model through the anti-inflammatory pathway. Although the antidepressant effect of Praxol has been verified in the 6-OHDA-induced PD model, its mechanism might involve changes in hippocampal regeneration (Chiu et al., 2015). In addition, MK-801 (Diazolizepine; Singh et al., 2017) and D1 receptor agonist (Mishra et al., 2019) were also suggested to improve depression behavior in 6-OHDA-induced PD model by promoting hippocampal nerve regeneration. Other drugs that could improve depression behaviors in 6-OHDA-induced PD models included Agametine (Souza et al., 2018), progesterone (Casas et al., 2011), granulocyte colony stimulating factor (G-CSF; Prakash et al., 2013), β3-adrenergic receptor agonist (Sampaio et al., 2019), Pioglitazone (Bonato et al., 2018), guanosine (Marques et al., 2019), Ketamine (Vecchia et al., 2018), imipramine (Vecchia et al., 2018), neuropeptide-S (Szot et al., 2016), Rapamycin (Masini et al., 2018) and Apamin (a selective blocker of small conductance calcium-activated potassium channels; Chen et al., 2014). It is alarming that Agomelatine has the risk of aggravating rhythm disorder besides its antidepressant effect (Souza et al., 2018). Excitingly, transcranial direct current stimulation (Feng et al., 2020), deep brain stimulation (Furlanetti et al., 2015, 2016), electroacupuncture (Sun et al., 2016; Yu et al., 2020), and exercise therapy (Goes et al., 2014; Tuon et al., 2014; Hsueh et al., 2018) also demonstrated certain effects on the improvement of depression in 6-OHDA-induced PD models, and most of them had multiple targets.

## Rotenone and PD-Related Depression

With the in-depth epidemiological investigation of PD, more and more attention has been attracted to the relationship between environmental toxins, especially pesticides used in agriculture, and the development of PD, and Rotenone is one of them. Rotenone is an insecticidal flavonoid found in many legumes and has been used as fish poison in Peru. It was later found to have high lipophilicity and therefore easy to pass through the blood-brain barrier. Like MPTP, Rotenone leads to PD-like symptoms by inhibiting mitochondrial complex I and causing oxidative stress (Heinz et al., 2017). Also, it mediates the degeneration of dopaminergic neurons in the substantia nigra striatum caused by the accumulation of α-synuclein. Notably, rotenone leads to non-specific toxicity and motor impairment unrelated to nigral cell damage as well (Fleming et al., 2004; Klein et al., 2011). At present, Rotenone is often used to induce PD models in zebrafish and rodents, and the latter is mainly achieved by systemic injection or direct stereotactic injection. High-dose Rotenone effectively induces motor dysfunction but produces a significant lethal effect. Based on this, people have tried to modify its application to achieve a proper balance between efficacy and lethality. It is under this background that Rotenone-induced non-motor phenotypes of PD have also attracted much attention (Fontoura et al., 2017), among which the depression phenotype is one of the research hot spots.

### General State of PD-Related Depression Induced by Rotenone

As shown in Table 3 (Santiago et al., 2010; Morais et al., 2012; Noseda et al., 2014; Zaminelli et al., 2014; Shin et al., 2017; Wang et al., 2017; Madiha and Haider, 2019), there are less than 10 reports on Rotenone-induced PD-related depression by now, with most reports using rats as the model animal (6/8), and one report each with mouse and zebrafish. The number of reports of each administration route of Rotenone were: brain stereotactic injection (3/8), intraperitoneal injection (2/8), subcutaneous injection (2/8), and addition in water (similar to environmental exposure; 1/8). And the dosage of Rotenone was different in each report, even with the same administration route. As predicted, the brain areas affected were mostly the hippocampus and striatum, and in one article the dorsal raphe nucleus was affected (Shin et al., 2017). In terms of behavioral evaluation, due to the restriction in the living environment of zebrafish, the depression-like behavioral changes could only be evaluated by limited methods, which was the dark box test in this study (Wang et al., 2017). For the behavioral evaluation of rats and mice induced by rotenone, only SPT and FST, but no TST or ST were reported. In addition, the depression-like behavioral changes mostly occurred after the manifestation of dyskinesia, of course, occasionally accompanied by anxiety, olfactory disorders, and other non-motor symptoms. In addition, some studies have used a compound model of rotenone combined with rapid eye movement sleep deprivation (Noseda et al., 2014).

**Table 3:** Summary of animal models of PD-related depression induced by Rotenone.

Number	Reference	Model animal	Gender	Administration route	Dosage of Rotenone	Assessment of depression behavior
1	Santiago et al. (2010)	280–320 g Wistar rats	Male	stereotactic injection (bilateral SN)	12 μg/side	ModifiedFST↑, SPT↓
2	Madiha and Haider (2019)	15–200 g Albino-Wistar rats	/	s.c.	1.5 mg/kg/day for eight consecutive days	FST↑, SPT↓
3	Morais et al. (2012)	200–230 g Wistar rats	Male	i.p.	2.5 or 5 mg/kg/day for 10 consecutive days	Modified FST↑, SPT↓
4	Zaminelli et al. (2014)	12-week 290–330 g Wistar rats	Male	i.p.	2.5 mg/kg/day for 10 consecutive days	Modified FST↑
5	Wang et al. (2017)	5–7 month wild-type Danio rerio	Male	in water	2 μg/L for 4 weeks	Dark box test
6	Noseda et al. (2014)	28–320 g Wistar rats	Male	stereotactic injection (bilateral SN)	12 μg/side	Modified FST↑
7	Chen et al. (2017)	8-week ICR mice	Male	stereotactic injection (unilateral lateral cerebroventricle)	0.2 μmol/kg, 5 μl	SPT↓, FST↑
8	Shin et al. (2017)	9-week Sprague-Dawley rats	Male	s.c.	3.0 mg/kg/day for 10 consecutive days	FST↑

*SN, substantia nigra; i.p., intraperitoneal injection; s.c., subcutaneous injection; FST, forced swimming test; SPT, sucrose preference test.↑, The increased immobility time;↓, The presence of anhedonia*.

### Possible Mechanism of PD-Related Depression Induced by Rotenone

Like the small number of total reports, there were few literatures on the mechanism of PD-related depression induced by Rotenone, which generally involved oxidative stress or neurotransmitter changes, whereas no specific signaling pathways or neural loops were involved.

### Possible Interventions in PD-Related Depression Induced by Rotenone

In the limited number of reports, curcumin (Madiha and Haider, 2019), ibuprofen (Zaminelli et al., 2014), the melatonin MT2 receptor-selective antagonist 4-P-PDOT (Noseda et al., 2014), and exercise therapy (Shin et al., 2017) were found to improve the depression symptoms in the Rotenone-induced PD model.

## Lipopolysaccharide (LPS) and PD-Related Depression

LPS is an endotoxin released from the destroyed outer cell wall of Gram-negative bacteria. LPS can activate epithelial cells, mononuclear macrophages, and endothelial cells to synthesize and release many inflammatory mediators and cytokines through a variety of signaling pathways, thus triggering a series of reactions in the body (Morris and Li, 2012). In general, a high concentration of LPS causes an extensive and strong inflammatory response, while an appropriate concentration can initiate a moderate immune response to enhance immune function. With the increasing attention on the role of neuroinflammation in the development of PD (Vivekanantham et al., 2015), the successful establishment of animal PD models induced by LPS (Joers et al., 2017), and the in-depth exploration of the function of LPS in depression, the reports of LPS in the study of PD-related depression are also gradually increasing.

### General Status of PD-Related Depression Induced by LPS

As shown in Table 4, there were fewer reports of PD-related depression induced by LPS. In the six studies we retrieved, the PD models were mainly induced by intraperitoneal injection of LPS in mice (4/6), with variance in sex and dose. The remaining two articles reported LPS-induced depression in PD rats, one by intraperitoneal injection and the other by brain stereotactic injection. Interestingly, despite the limited number of articles, several studies attempted low-dose LPS induction that caused only depression but no motor damage. In addition, FST was used in all six studies, either alone or in combination with SPT, SP, or TST. The only other non-motor symptom manifested was anxiety-like behavioral changes in these models.

**Table 4:** Summary of animal models of PD-related depression induced by lipopolysaccharide (LPS).

Number	Reference	Model animal	Gender	Administration route	Dosage of LPS	Assessment of depression behavior
1	Chen et al. (2017)	8-week ICR mice	Male	i.p.	0.8 mg/kg	FST↑, SPT↓
2	Hritcu and Gorgan (2014)	3-month 200 ± 50 g Wistar rats	Male	stereotactic injection (unilateral SN)	3 μg/kg, 10μg/kg	FST↑
3	Ventorp et al. (2017)	200 g Wistar rats	Male	i.p.	1 mg/kg	FST↑
4	Lieberknecht et al. (2017)	60–120-day 40–55 g Swiss mice	Female	i.p.	0.1 mg/kg	FST↑, ST↓
5	Yuan et al. (2020)	20–25 g C57BL/6 mice	Male	i.p.	0.83 mg/kg	FST↑, TST↑
6	Yang et al. (2016)	12-week adult mice	/	i.p.	330 μg/kg	FST↑, TST↑
7	Santiago et al. (2010)	Male Wistar rats	Male	stereotactic injection	2 μg/side	SPT↓, Modified FST↑

*i.p., intraperitoneal injection; SN, substantia nigra; FST, forced swimming test; SPT, sucrose preference test; ST, splatter test; TST, tail suspension test.↑, The increased immobility time; ↓, The presence of anhedonia*.

### Possible Mechanism of PD-Related Depression Induced by LPS

As LPS induces a strong inflammatory response, the mechanism of PD-related depression induced by LPS is generally considered to be related to neuroinflammation and neuroimmunity. As a matter of fact, neuronutrition and oxidative stress may also play important roles, especially the changes in the BDNF signaling pathway.

### Possible Interventions in PD-Related Depression Induced by LPS

Based on the pro-inflammatory role of LPS, in theory, anti-inflammatory therapy should be the first choice of intervention for LPS-induced PD-related depression. However, among the interventional studies, there was only one literature that suggested improved depression symptoms in the LPS-induced PD model “simply” through anti-inflammatory therapy using not conventional drugs but dopamine receptor agonist Praxol (Lieberknecht et al., 2017). In addition, BCG vaccination could improve LPS-induced PD-related depression through immune regulation, which was a far-fetched evidence of the interventional efficacy of anti-inflammation on PD-related depression (Yang et al., 2016). Although Besarotene was reported to play certain roles against LPS-induced PD-related depression through anti-inflammation, this effect might be mainly related to the restoration of dysregulated CREB/BDNF/ERK signaling pathway (Yuan et al., 2020). More surprisingly, the anti-depressant effect of Exendin-4, a glucagon-like peptide 1 (GLP-1) receptor peptide agonist, does not involve anti-inflammation, but to a large extent related to its effect on the synthesis or metabolism of dopamine (Ventorp et al., 2017). As previously mentioned, oxidative stress is one of the mechanisms of LPS-induced PD-related depression, Resveratrol might effectively intervene the depression symptoms in the LPS-induced PD model through blockade of oxidative stress (Chen et al., 2017).

## Others: Paraquat and Lactocin and PD-Related Depression

In addition to the above neurotoxins, paraquat, and lactocin were also reported to induce depression in PD animal models. Since there were only two reports of depression induced by paraquat and one report by lactocin, they are discussed together here.

### Paraquat and PD-Related Depression

Paraquat (N,N’-dimethyl-4,4’-bipyridine) is an herbal compound with a similar structure to MPP^+^, and is used as a herbicide in agriculture. It is specifically toxic to many organs of the human body. It crosses the blood-brain barrier into brain dopaminergic neurons unreliable on DAT but may be transported by neutral amino acid transporters in an age-dependent manner. The mechanism of paraquat-induced cell death is also different from that of MPP^+^ and rotenone, because it cannot effectively block mitochondrial complex I, but involves the mitochondrial endogenous pathway (Fei et al., 2008). As paraquat can induce the accumulation of α-synuclein and the degeneration of dopamine neurons in substantia nigra, it was also used to establish PD animal models (Bastías-Candia et al., 2019). However, the phenotypes of models constructed by different studies were not consistent, and even contradictive in some cases, which more or less restricted its application. With the accumulating evidences that combined the use of paraquat with other compounds (such as manganic manganese) could cause more severe motor damage, its application in the establishment of PD models has regained new attention. Of course, its effects on PD-related depression and other non-motor disorders are also worthy of study.

Between the two studies of paraquat-induced PD models, one of them successfully induced PD-related depression by combined use of paraquat with mancozeb (Hou et al., 2019). Three-month-old male C57BL/6J mice received intraperitoneal injection of paraquat (10 mg/kg) + mancozeb (30 mg/kg) twice a week for 4 weeks. Depression-like behavior was verified by FST. At the same time, this model also displayed non-motor functional changes such as constipation, learning difficulty, and memory impairment. In addition, this study inversely proved through pharmacological approaches that depression symptoms in the PD model might not be related to locus coeruleus and norepinephrine. In another report, a miniature osmotic pump was implanted in the subscapular area to introduce paraquat at a rate of 0.7 mg/day for 28 days, which successfully induced motor disorders as well as depression and anxiety (Campos et al., 2013).

### Lactocin and PD-Related Depression

Lactocin, a proteasome inhibitor, is derived from the metabolites of Streptomyces in soil and widely exists in the environment (Kisselev and Goldberg, 2001). When susceptible people ingest Lactocin through daily contact and diet, it can cause functional deficiency of the ubiquitin-proteasome system by inhibiting the 20S/26S proteasome function, leading to the accumulation of abnormal proteins, and inducing a variety of diseases including PD. This is mainly because it can cause synuclein accumulation similar to the effect of paraquat, selectively kill dopaminergic neurons in the substantia nigra-striatum system, and decrease the level of striatal dopamine, resulting in motor dysfunction (Niu et al., 2009).

Some researchers have successfully constructed PD animal models using Lactocin, which showed typical PD symptoms that progressively aggravated (Harrison et al., 2019). There was no toxin accumulation in the animals and did not cause animal death or paralysis. However, some researchers believed that this model has poor repeatability which might be due to the poor absorption of Lactocin by the brain tissue due to the low solubility of Lactocin before administration or due to the formation of precipitation after injection, which could easily lead to the failure of the experiment. But it is still a method worth exploring if this problem can be solved. Recent studies have optimized the dose of Lactocin or used it in combination with other drugs such as LPS and observed a more significant phenotype of PD (Deneyer et al., 2019). However, the research on non-motor symptoms such as depression in Lactocin-induced models is still rarely reported. Only one study detected signs of depression for the first time in a preclinical experimental model of PD rat induced by intranasal administration of Lactocin (Ekimova et al., 2018).

## Reflection and Conclusion

**Figure 1:**
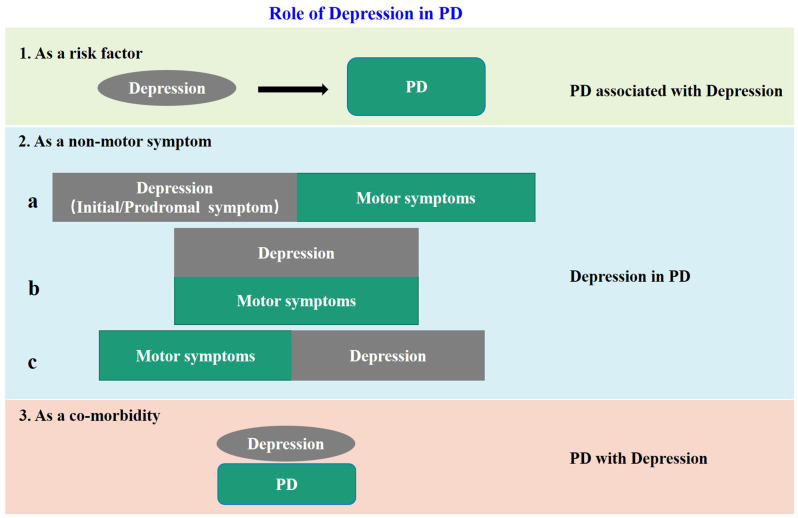
The model diagram of the relationship between depression and PD. In case 1, depression is a risk factor for PD; in case 2, depression is a non-motor symptom of PD, and there are three sub cases, that is, it appears before motor symptoms as a prodromal symptom: (a) occurs simultaneously with motor symptoms; (b) and appears after motor symptoms; and (c) in case 3, it appears as an independent disease and comorbidity of PD.

The negative influences of depression in PD patients are more and more recognized. However, because of the complex relationship between depression and PD, the definition and classification of “PD-related depression” are not uniform at present. Accumulating clinical evidences show that depression is not only a risk factor for PD, but also a non-motor symptom, or even a co-morbidity of PD. When regarded as a non-motor symptom of PD, depression can appear synchronous with, before or after the motor impairment. Based on previous reports and our own clinical experience, we summarized the above issues in Figure 1, and put forward some thoughts on PD-related depression. Firstly, all the clinical cases of “depression” and “PD” encountered at present can be collectively referred to as “depression related to PD”. When depression is “as a risk factor” of PD, it can be called “PD associated with depression”, when “as a non-motor symptom” can be called “depression in PD”, and when “as a co-morbidity” can be called “PD with depression.” Secondly, it is unfortunate for individual patients no matter which of the above situations, therefore proper identification and treatment are most important. But from the aspect of community prevention and control, the situations shown in “1” and “2a” in Figure 1 are the key nodes to achieve early detection and early intervention to avoid or delay subsequent dopaminergic neuronal damage, and to achieve proper neuroprotection. Thirdly, in the current clinical prevention and treatment guidelines for PD, treatment of depression is mostly based on the treatment of motor impairments. This is inevitable because as required by the current evidence-based medical principles, a patient with depression but without motor symptoms cannot be treated as PD-related depression in the first place even if the patient later developed PD symptoms. To resolve this dilemma, on one hand, it is necessary to launch large-scale prospective cohort studies in the target population, for example, the “Taicang cohort study” led by Professor Chun-feng Liu of our research team, which is currently carried out in Taicang, China. On the other hand, it is necessary to establish a suitable preclinical animal model for basic research. This is because when the neurobiological nature of PD-related depression is not yet fully understood, the use of animal models is still of great significance to improve our understanding of the etiology, pathophysiology, and molecular mechanism of PD. Besides, the research time for constructing animal models is relatively short. It will undoubtedly accelerate the research of PD-related depression if we can establish a suitable animal model.

At present, many animal models of PD have been recognized and widely used, which can be divided into two categories (Blesa et al., 2012): one is by damaging dopaminergic neurons using natural (e.g., Rotenone, LPS, Lactocin) or synthetic neurotoxins (e.g., MPTP, 6-OHDA, paraquat), and the other is by using transgenic animals containing PD-related gene variants. At present, the transgenic PD model is actually limited to transgenic mice with synaptic dopamine depletion, and the behavioral phenotype is usually not obvious. In addition, although there are several gene variants associated with the development of non-motor symptoms in PD patients (e.g., LRRK2, SNCA, Parkin, and VPS35), only a few studies have explored their effects on depression-like behavior in mice (Fontoura et al., 2017). It is worth noting that some studies have constructed a depression-PD co-morbidity animal model using Pitx3 (a risk gene of PD) deficient mice (Kim et al., 2014) or mice treated intraperitoneally with reserpine (Skalisz et al., 2002). Another study found Reserpine could be used to establish the depression-PD association animal model. An ideal PD-related depression animal model has four characteristics: (1) with significant and detectable depress phenotype; (2) with typical and detectable PD-specific motor impairment that aggravates progressively with aging; (3) simulates the clinical pathophysiological evolution of patients with PD-related depression; and (4) reproduces the progressive changes of neurotransmitters in different brain regions of patients with PD-related depression. Regardless of whether the above co-morbidity models meet these characteristics, they still fail the “early detection and early intervention” requirement in situations “1” and “2a” (Figure 1). Although situation “1” has been achieved through initial induction of PD by stress, followed by induction of motor impairment by neurotoxin (Hemmerle et al., 2012), this protocol might actually complicate the situation, because more and more studies found that the PD model induced by neurotoxin alone exhibited depression-like behavior. And as we have previously concluded, after administration of neurotoxin, depression changes can occur before motor damage, which simulates the “2a” situation, and may even partially represent the “1” situation. In summary, under the current condition of lacking spontaneous PD-related depression models, the PD models induced by neurotoxins may be used at this stage or even for a long time in the future. Due to the differences in the phenotype of PD-related depression induced by different neurotoxins, it is a difficult problem for the majority of researchers at present to select the appropriate neurotoxin, method, and dose in the construction of PD-related depression models. Facing this predicament, researchers compared the effects of different neurotoxins on PD-relation depression models. For example, a study used rats matched with age, body weight, and sex and induced by stereotactic injection of MPTP, 6-OHDA, Rotenone, and LPS in bilateral substantia nigra (Santiago et al., 2010). All but LPS induced depression-like behavior, and 6-OHDA had the best phenotype. Another study compared the non-motor symptoms of three PD models (Campos et al., 2013): 6-OHDA (8 or 6 μg, stereotactic injection of bilateral substantia nigra pars compacta), paraquat (micro-osmotic pump implanted in the subscapular area at the rate of 0.7 mg/day for 28 days) and α-synuclein overexpression transgenic model, and found that 6-OHDA and paraquat-induced depression-like behavioral changes in model animals. From the above two comparative studies, it is not difficult to find that the 6-OHDA might have the best effect in inducing PD-related depression. From Tables s 1–4 we can also find that there were the largest number of reports of 6-OHDA-induced PD-related depression, and unilateral MFB brain stereotactic injection was the first choice of administration route. However, the specific dosage of 6-OHDA should be determined according to the actual conditions of animals, as there is no unified standard at present, which inevitably leads to a variety of study results. What we can do is to carefully analyze and identify the genuinely useful information.

In addition to the usage of different types of neurotoxins and their dosage, the success rate of model construction also needs to be considered. At present, the incidence of PD–related depression induced by neurotoxins has only been reported in MPTP-induced models. Of course, the conditions of animals such as species, sex, age, bodyweight, etc., also need to be concerned, especially the sex factor itself has an impact on depression and PD in humans (both occur more in women than in men), which have been particularly emphasized in some studies (Sullivan et al., 2014; Schamne et al., 2018). Another factor that requires comprehensive analysis is the behavioral evaluation of model animals. As we have summarized in Tables s 1–4; a single behavioral test was used in many neurotoxin-induced PD-related depression models, which would affect the extrapolation of the results to certain extents. For example, FST, TST, and ST all involve “movement”, therefore, it is necessary to rule out the interference of motor impairment. On the other hand, as the SPT test involves the taste function as well as the motor function of the animal, the interference of both factors should be excluded for proper evaluation of depression behaviors. At the same time, different behavioral testing operations are different (specific operations are not described here due to limited space and specific literature can be searched), and their sensitivities are also different. Therefore, we suggest that multiple behavioral tests should be used for cross-validation, and even introduce a rescue experiment for forward and backward validation. Of course, if the model modulates situations “1” and “2a”, the interference of motor factor is neglectable, but it is important to truly rule out motor dysfunction through movement experiments. Further, there is also an important challenge that the results of these depressive behavior tests have not been convincingly coupled to neuropathological changes (Nestler et al., 2002).

Last but not least, the purpose of establishing a PD-related depression animal model is to facilitate the study of the pathogenesis and prevention and treatment strategies of PD-related depression. At present, the research on the mechanism of PD-related depression in neurotoxin-induced animal models is mostly originated from the hypothetical factors of the development of PD-related depression, such as the neurotransmitters, neuronutrition, nerve regeneration, nerve inflammation, and oxidative stress. However, there are very few studies on the involvement of the nerve loop, which is the focus of clinical study, in the PD-related depression model induced by neurotoxin. Moreover, the intrinsic differences of toxins in generic models may also play a role in the mechanisms underlying the depression phenotype. It is worth mentioning that their effects on the neurotransmitter system are different. For example, both MPTP and 6-OHDA can act on the mitochondrial complex in the cell and produce cytotoxicity, resulting in the disorder of neurotransmitters in the dopamine system, but it seems that 6-OHDA can also cause other neurotransmitter changes, such as 5-HT (Zhang et al., 2010). More interestingly, rotenone increased the level of 4-hydroxy-3-methoxyphenylacetic, a norepinephrine metabolite, in the striatum (Thiffault et al., 2000). These tips NA-5HT-DA cross-link in the neurotoxin PD models may be an important breakthrough to explore the mechanism of PD depression. As is known to all, structures define functions, so the structural alterations of depression-related brain areas (Remy et al., 2005), for example, the amygdala and hippocampus, in the neurotoxin PD model should also be concerned. With the continuous improvement of optogenetics and single-cell analysis technology, we believed that there will be a more and more precise exploration in this field in the future. In the meantime, it is encouraging that in the exploration of effective prevention and treatment of PD-related depression using neurotoxin-induced models, deep brain stimulation, electrostimulation, and exercise therapy have emerged besides pharmacological interventions. However, there has been no report using the noninvasive transcranial magnetic stimulation (TMS) that may be effective in the clinical treatment of PD-related depression patients. We look forward to future research that uses TMS in animal models of PD-related depression induced by neurotoxin.

In conclusion, in order to better reveal the relevant changes in the development of PD-related depression and its possible mechanism, and to develop targeted prevention and treatment strategies, it is the key point in current studies to establish a preclinical animal model that is generally in line with the three validity criteria [predictive validity (the ability of the model to predict certain clinical events), face validity (phenomenological similarities between models and clinical conditions), and construct validity (similarities between potential mechanisms of animal behavior and psychological or neurobiological mechanisms under clinical conditions)] (Skalisz et al., 2002) and infinitely close to the four characteristics of ideal PD-related depression models. Neurotoxin-induced PD-related depression models are worthy of further improvement and optimization.

## Author Contributions

S-ZW: conception and design. CR and Y-QJ: administrative support. X-YS, J-HW, and Y-QJ: provision of study materials. X-YY, L-NG, and Y-KM: collection and assembly of data. CR and S-ZW: data analysis and interpretation. All authors: manuscript writing and final approval of manuscript. All authors contributed to the article and approved the submitted version.

## Conflict of Interest

The authors declare that the research was conducted in the absence of any commercial or financial relationships that could be construed as a potential conflict of interest.

## Publisher’s Note

All claims expressed in this article are solely those of the authors and do not necessarily represent those of their affiliated organizations, or those of the publisher, the editors and the reviewers. Any product that may be evaluated in this article, or claim that may be made by its manufacturer, is not guaranteed or endorsed by the publisher.
